# Statistical models for analyzing count data: predictors of length of stay among HIV patients in Portugal using a multilevel model

**DOI:** 10.1186/s12913-021-06389-1

**Published:** 2021-04-21

**Authors:** Ahmed Nabil Shaaban, Bárbara Peleteiro, Maria Rosario O. Martins

**Affiliations:** 1 Department of Global Health and Tropical Medicine, Institute of Hygiene and Tropical Medicine, NOVA University of Lisbon, Rua da Junqueira N°100, 1349-008 Lisbon, Portugal; 2 EPIUnit - Instituto de Saúde Pública, Universidade do Porto, Porto, Portugal; 3 Departamento de Ciências da Saúde Pública e Forenses e Educação Médica, Faculdade de Medicina da Universidade do Porto, Porto, Portugal

**Keywords:** Length of stay (LOS), Count data analysis, HIV, Hospital performance, Quality indicator, Random - effects model, Multilevel model#

## Abstract

**Background:**

This study offers a comprehensive approach to precisely analyze the complexly distributed length of stay among HIV admissions in Portugal.

**Objective:**

To provide an illustration of statistical techniques for analysing count data using longitudinal predictors of length of stay among HIV hospitalizations in Portugal.

**Method:**

Registered discharges in the Portuguese National Health Service (NHS) facilities Between January 2009 and December 2017, a total of 26,505 classified under Major Diagnostic Category (MDC) created for patients with HIV infection, with HIV/AIDS as a main or secondary cause of admission, were used to predict length of stay among HIV hospitalizations in Portugal. Several strategies were applied to select the best count fit model that includes the Poisson regression model, zero-inflated Poisson, the negative binomial regression model, and zero-inflated negative binomial regression model. A random hospital effects term has been incorporated into the negative binomial model to examine the dependence between observations within the same hospital. A multivariable analysis has been performed to assess the effect of covariates on length of stay.

**Results:**

The median length of stay in our study was 11 days (interquartile range: 6–22). Statistical comparisons among the count models revealed that the random-effects negative binomial models provided the best fit with observed data. Admissions among males or admissions associated with TB infection, pneumocystis, cytomegalovirus, candidiasis, toxoplasmosis, or mycobacterium disease exhibit a highly significant increase in length of stay. Perfect trends were observed in which a higher number of diagnoses or procedures lead to significantly higher length of stay. The random-effects term included in our model and refers to unexplained factors specific to each hospital revealed obvious differences in quality among the hospitals included in our study.

**Conclusions:**

This study provides a comprehensive approach to address unique problems associated with the prediction of length of stay among HIV patients in Portugal.

## Background and introduction

Length of stay (LOS) is a key instrument to assess the quality of care in light of recent attempts to control the increasing costs of health care services [1–3]. The number of days a patient stays at the hospital represents a good illustration of resources utilized during the in-patient hospitalization [4]. Shorter inpatient stays reduce hospital resources consumption; hence, decrease the related health expenditure [5]. However, although hospitals are acclimating to clinical and financial standards induced by policy reforms to reduce length of in-patient care, hospitals’ quality tends to vary widely [4]. In our previous paper [3], we shed light on the importance of length of stay as a quality indicator in Portugal. Determining the factors that may push LOS further may add to efforts in controlling unnecessary days of admissions, planning resources allocation, and customizing appropriate interventions [4, 5]. Unfortunately, the use of common statistical techniques, particularly the ordinary least squares (OLS) and the logistic regression, to analyze the predictors of LOS as a count variable with overdispersion can violate the assumptions behind each technique, leading to biased estimates that do not precisely reflect the observed data [3, 6, 7] (for an informative overview of count distribution see Atkins, Baldwin, Zheng, Gallop, & Neighbors, 2013 [8]). Relatively recently, several statistical models have been generated to analyze data with count nature [3, 9, 10].

The first model to analyze count outcomes is the Poisson regression model (PRM) [6, 11, 12]. This model is based on Poisson distribution has two restrictive assumptions [6, 12, 13]. First, the variance of the count outcome is equal to the mean. The second assumption is that occurrences of events are independent of each other [6, 12]. However, in practice, these assumptions are usually violated [6, 12], and count variables tend to have a conditional variance that often exceeds the conditional mean, which is known as “overdispersion” [6, 14]. Using the PRM to analyze outcomes in which one of these two assumptions is violated may result in biased data with underestimated standard error [12].

The second model is the negative binomial regression model (NBRM) that attempted to overcome the above-mentioned limitations in the Poisson distribution and has proven to properly represent the observed counts than the Poisson distribution [6, 15]. Accordingly, unlike the PRM, this distribution does not require the mean and variance of the count outcome to be equal [6, 12]. Additionally, the previously mentioned assumption of independence of events required for PRM is no longer mandatory in the NBRM since it assumes that events can be repeated, given the influence of individual differences on the probability of an event to occur [6, 12, 13].

Two other alternatives count models are the Zero-Inflated Count Models: zero-inflated Poisson (ZIP) and zero-inflated negative binomial (ZINB). These models had been developed to overcome circumstances in which the origin of overdispersion is due to excessive zero counts. These kinds of distributions assume that the zero counts originate from two different sources and can be classified into two groups [16]. The first group is the “structured zeros” in which there is no chance to go beyond zero [16, 17]. For example, some HIV patients may have been admitted to a hospital and discharged on the same day (same-day separation) because they do not need further treatment, and hence, counted as a zero-length of stay (in days). This phenomenon of same-day separation can be explained by the fact that advancements in healthcare services, medical treatment, and technologies have allowed health facilities to provide more efficient services and improve patients’ outcomes [18]. As a result, a steady decrease in length of stay and an increase in the probability of same-day separations can be observed [18]. These hospitalizations with same-day separations (LOS = 0) which partially constitute the zero counts should be distinguished from the overnight stays (LOS = 1) for resource allocation purposes. On the other hand, the remaining patients with zero count are classified as the “sampling or random zero” group [16, 17] as they can be admitted for more than zero days, e.g. a patient who could have been hospitalized for a non-zero number of days but decides to exit against medical advice before completing overnight stay and hence counted as zero number of days. The first two count models, Poisson and negative binomial are not sufficiently fit for this kind of data since they may fail to address the excess zeros that arise from two different data generating methods, and hence, may induce overestimated variance of model parameters [5]. After considering the excessive zero problem, the zero-inflated techniques generate two regression models. The first model predicts the occurrence of the count, while the second regression model predicts the frequency of occurrence of this count [6, 11]. The zero-inflated model selection, whether ZIP or ZINB, is determined by the sort of overdispersion. If the excessive number of zeros generates the overdispersion, then the ZIP is more appropriate to model count data [6, 11]. On the other hand, if the overdispersion is caused by factors not related to the excessive number of zeros, then the ZINB model is more suitable [6, 11].

However, in some specific contexts in which hierarchically structured data are the norm, as in the case of a patient in hospital multilevel data, the use of the above-mentioned ordinary count models may violate the assumption of independence of variance of the ordinary count regression [5, 19]. The multilevel models (or the random-effects models) are becoming increasingly popular in the social, behavioral, and medical sciences in which hierarchically structured data [20, 21]. In Diagnosis Related Group (DRG) data, as in our study, patients tend to cluster within hospitals based on their preferences such as neighborhood, trust in a specific physician, or hospital, and hence violating the assumption of independence of variance of the ordinary count regression [5, 19]. Accordingly, multilevel modelling is more appropriate than ordinal count regression when dealing with data arranged in a hierarchical structure, such as patients nested within hospital two-level data [22–24]. Random-effects models can help in identifying outstanding hospitals and assess hospital quality accordingly [18]. Additionally, ignoring the existence of within-hospital clustering, as in-patient level analysis with no hospital random effect terms generates serious technical problems [20]. Disregarding this clustering will lead to an increase in the number of independent observations at the hospital level, thus underestimating the standard errors of regression coefficients at the hospital’s level [20, 25, 26]. Therefore, the random-effects model is more relevant to analyze the impact of a set of predictors on LOS while controlling for variations in hospitilizations and hospital characteristics [25–27].

In Portugal, hospitalizations among HIV/AIDS patients account for the highest average LOS and represent a substantial economic burden, being classified as the second major diagnosis category [3, 28]. Morever, it is important to understand whether the zero-inflated data can affect selecting the appropriate count regression model. Previous studies among HIV hospitalizations in Portugal either used logistic regression [28] or Poisson regression [29] to analyze a count variable with overdispersion. Accordingly, unlike other studies, this study aims to examine the predictors of LOS using the best count fit model through comprehensive comparisons between the different count models using the national admissions database among HIV patients in Portugal.

## Methods

### Data collection and source

The present analysis is based on data collected as part of the national registry of discharges among the Portuguese National Health Service (NHS) facilities. We reviewed hospitalization records for all HIV/AIDS patients admitted in Portugal between 1st January, 2009, and 31st December, 2017. Each record corresponds to a discharge episode and contains information collected while the patients were admitted to the hospital, such as type of admission, principal diagnosis, secondary diagnoses, procedures, region, age, sex, and discharge status. These data are anonymous, refers to the Diagnosis Related Groups (DRGs), and were obtained through the Central Health System Administration (ACSS) [30]. The DRGs were first introduced in Portugal through a pilot study in 1984, and since 1989 a systematic collection of DRGs applies to NHS hospitals [19, 31, 32]. Implementing DRGs in Portugal aimed to increase transparency and vindicate the allocation of resources to NHS hospitals by connecting inpatient care resources to hospital outcomes [31]. Since August 2006, there is only one non-modified version of DRGs in use in Portugal, knowing as All Patients DRGs version 21 (AP–DRGs version 21) [32]. This version is implemented in all Portuguese NHS hospitals and applies to all inpatients and ambulatory surgery with an exception for patients treated in psychiatric and rehabilitation care settings [31]. The AP–DRGs version 21 includes 669 DRGs under 25 Major Diagnostic Categories (MDCs), and each MDC refers to one organ or physiological system, with the MDC 24 corresponding to HIV Infection [31]. Since 1984, diagnoses and procedures within the DRGs system have been coded using the International Classification of Diseases, Ninth Revision, Clinical Modification (ICD-9-CM), and since 2016 they have been coded using ICD-10-CM [31, 33].

The collection and validation of the DRG database in Portugal are carried out through several internal and external auditing steps to ensure high-quality data, a criterion necessary for any DRG system [31]. Coding in Portugal is first standardised and performed by physicians with specific training in coding [31]. An internal auditor assigned by each hospital supervises the data collection and the coding process. Afterward, the external coding auditing process starts with trained physicians supervised by a senior manager from the ACSS to assist and monitor the internal auditing process [31]. Moreover, this external auditing team is authorized to carry out visits for the included hospitals and validate patients’ records to ensure if the DRG coding and classification have been done correctly [31]. It is also important to mention that the DRG clinical coding process is carried out through computer software to identify data errors and discrepancies in hospital records [31]. Afterward, this software delivers data regarding the mean number of codes per record, the proportion of incorrect codes and coding errors, together with notifying the operator of deficient information within the records such as duplications, undefined primary diagnosis, or atypical lengths of stay [31]. We also used a unique fictional code included in the data for data validation that allows determining how many episodes correspond to the same user in the same institution, hence avoid duplicates. This fictional code does not identify the user or allow its identification afterward.

### Study participants

A total of 26,505 discharges among patients aged 18 years or older were included in the study. We considered only discharges classified under MDC created for patients with HIV infection (MDC 24). For the purpose of this study, data about discharges with HIV/AIDS as a main or secondary cause of admission were analyzed. Primary and secondary diagnoses and procedures were coded according to the International Classification of Diseases. Nineteen secondary diagnoses and up to 20 procedures were considered in this study.

### Dependent variable

The dependent variable in our study is length of stay (LOS) which is defined as the number of days between in-patient admission and hospital discharge.

### Data analysis

**Fig. 1:**
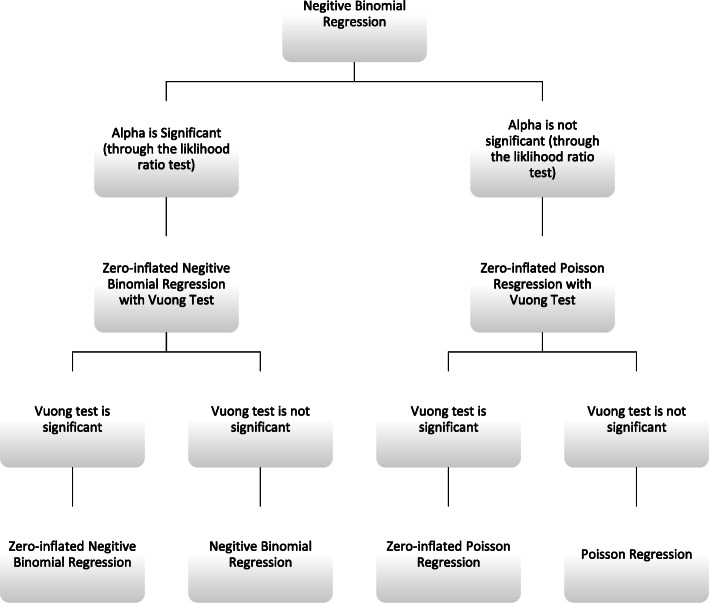
Flowchart for selecting a count regression model in analyzing length of stay among HIV patients in Portugal

Descriptive and univariate analyses were carried out. A *p*-value ≤0.05 was considered to be significant. To select the best count model, we applied several strategies that included three separate phases. The first phase in our analysis was to test the overdispersion of our count variable. Accordingly, the mean and the variance of LOS were calculated. In the second phase of our analysis, we included a flowchart (Fig. 1) to assist in deciding the most fitting count technique. By using STATA® software, we confirmed overdispersion, this time by using the likelihood ratio test to examine for overdispersion. We took the advantage that STATA® automatically calculates the likelihood-ratio (LR) whenever a negative binomial model is applied to examine if the dispersion parameter is equal to zero [6] (for more informative overview of the LR see Long, 1997 [34]). The following stage in the second phase of our analysis included the evaluation of the excessive zero counts, which is represented by the left half of our flowchart. We used Vuong test to examine any significant increase in the zero counts by considering the predicted probabilities of two count models [34].

In the third phase of our analysis, an exploratory analysis using the “countfit” estimation command in STATA® [35] was done to distinguish the corresponding fit of the different count models. Model performance and estimates of precision for each of the models were calculated. The command also generates the Akaike information criterion (AIC) and the Bayesian information criterion (BIC), which are estimators of the corresponding quality of statistical models [36, 37]. Each test estimates the quality of each of the four models compared to each of the other models. Hence, they provide an approach for model selection. Accordingly, we examined the model fit by comparing the AIC, BIC, and predicted and observed probabilities of each count outcome for each probability distribution. To show the effect of choosing a model in which its assumptions are violated, the ordinary linear regression model was included in our study for the purpose of comparison.

In our multivariable analyses, to assess the effect of covariates on LOS, information on patients, clinical characteristics, admissions’ characteristics were extracted from the hospital discharge database. The following potential determinants of LOS were considered: age (1 = “18–29″, 2 = “30–39″, 3 = “40–49″, 4 = “50–59″, 5 = “60–69″, 6=” > =70″), gender (0 = male, 1 = female), region (0 = Non-resident, 1 = Norte, 2 = Centro, 3 = Lisbon, 4 = Alentejo, 5 = Algarve), HIV/AIDS as a primary diagnosis (0 = no, 1 = yes), having national health coverage (SNS) (0 = no, 1 = yes), admission status (0 = elective, 1 = emergency), treatment classification (0 = medical, 1 = surgical), number of diagnoses (0 = “<=5″, 1 = “6–8″,2 = “9–12″, 3 = “> 12″), number of procedures (0 = “<=4″, 1 = “5–7″, 2 = “8–9″, 3 = “> 9″), in-hospital death (0 = no, 1 = yes), transferred admission (0 = no, 1 = yes), 30-day readmission (0 = no, 1 = yes), tuberculosis (TB) (0 = no, 1 = yes), Hepatitis B (0 = no, 1 = yes), Hepatitis C (0 = no, 1 = yes), Pneumocystis (0 = no, 1 = yes), Cytomegalovirus (0 = no, 1 = yes), Mycobacterial disease (0 = no, 1 = yes), Toxoplasmosis (0 = no, 1 = yes), Candidiasis (0 = no, 1 = yes), Kaposi’s sarcoma (0 = no, 1 = yes), history of recurrent pneumonia (0 = no, 1 = yes), weight loss (0 = no, 1 = yes), asymptomatic HIV (0 = no, 1 = yes). Since several hospitals have been merged in one hospital during the period between 2009 and 2017, we created a dummy variable (Merge) to categorize hospitals according to the merging status (0 = not merged, 1 = merged) to be able to study the effect of merging on hospital quality. We created a dummy variable for the years of admission in which years before the financial crisis and bailout in Portugal were coded as 0 (2009, 2010, and 2011), while years after the financial bailout were coded as 1 (2012, 2013, 2014, 2015, 2016 and 2017).

After choosing the most appropriate count technique, and in addition to the fixed patient and clinical related factors, we incorporated a random hospital effects term to the negative binomial model to examine the dependence between observations within the same hospital. Multiple comparisons of hospital effects were done by constructing 95% confidence intervals (CI) for random-effects.

All analyses were conducted with STATA®, version 13 (StataCorp LP, College Station, Texas, USA), and MLwiN®, version 3.04 (University of Bristol, Bristol, UK). MLwiN is a specialized software package for fitting multilevel models [38].

## Results

Table 1 summarizes the main characteristics of the study population. The study population was mostly composed of males (71.3%), with a median age of 44 years (interquartile range (IQR): 38–53)), with more than half living in Lisbon (52.9%), and almost 3% not being registered in the national health system. Admissions during 2009 accounted for 14.6% of the total admissions representing the highest admission rate, while admissions during 2017 accounted for 6.5% of admissions representing the lowest admission rate. A descending trend in the frequency of admissions was observed through the years. Most of the hospital admissions were preceded by an emergency (83.3%), with 12.0% of them resulting in the death of the patient, 6.4% were discharged to another health facility, and 4.1% exit against medical advice. In 9.5% of the admissions, the patient had to be transferred to another hospital, and in 11%, the patient had been readmitted within 30 days of the last discharge. Most episodes were classified as medical (94.2%). The median number of procedures per episode was 8 (IQR: 5–12), whereas the median number of diagnoses was 7 (IQR: 5–10). HIV was the primary diagnosis in 67.6% of the hospital admissions, while 6.6% of the patients had asymptomatic HIV infection at hospital admission. Hepatitis C was the most frequent co-infection (25.1%), followed by tuberculosis (7.3%), pneumocystosis (7.0%), mycobacterial infections (6.5%), Hepatitis B (4.3%), and cytomegalovirus (2.5%).

**Table 1:** Characteristics of the study sample (*N* = 26,505)

	N	%
**Gender**		
Male	19,011	71.73
Female	7494	28.27
**Age**		
18–29	1436	5.42
30–39	6801	25.66
40–49	9375	35.37
50–59	5205	19.64
60–69	2303	8.69
> =70	1385	5.23
**Region of residence (NUTS II)**		
Non Resident	357	1.35
Norte	6453	24.35
CENTRO	3806	14.36
LISBON	14,033	52.94
ALENTEJO	326	1.23
ALGARVE	1530	5.77
**Having SNS** ^**a**^		
Yes	756	2.85
No	25,749	97.15
**Year**		
2009	3863	14.57
2010	3735	14.09
2011	3576	13.49
2012	3537	13.34
2013	3146	11.87
2014	2502	9.44
2015	2223	8.39
2016	2189	8.26
2017	1734	6.54
**HIV as a primary diagnosis**		
No	8586	32.39
Yes	17,919	67.61
**Asymptomatic HIV**		
No	24,758	93.41
Yes	1747	6.59
**Hepatitis B**		
No	25,357	95.67
Yes	1148	4.33
**Hepatitis C**		
No	19,843	74.87
Yes	6662	25.13
**Associated TB infection**		
No	24,581	92.74
Yes	1924	7.26
**Pneumocystis**		
No	24,654	93.02
Yes	1851	6.98
**Cytomegalovirus**		
No	25,853	97.54
Yes	652	2.46
**Mycobacterium disease**		
No	24,779	93.49
Yes	1726	6.51
**Candidiasis**		
No	22,206	83.78
Yes	4299	16.22
**Toxoplasmosis**		
No	25,267	95.33
Yes	1238	4.67
**Kaposi Sarcoma**		
No	25,556	96.42
Yes	949	3.58
**Weight loss**		
No	25,649	96.77
Yes	856	3.23
**Number of procedures**		
< =5	7525	28.39
6–8	6720	25.35
9–11	5576	21.04
> 11	6684	25.22
**Number of diagnosis**		
< =5	9114	34.39
6–7	5514	20.80
8–10	5761	21.74
> 10	6116	23.07
**Emergency Admission**		
No	4424	16.69
Yes	22,081	83.31
**30-day Readmission**		
No	23,549	88.85
Yes	2956	11.15
**Hospital Death**		
No	23,316	88.0
Yes	3192	12.0
**Recurrent Pneumonia**		
No	26,326	99.32
Yes	179	0.68
**Mode of transfer**		
No transfer	23,995	90.53
Transferred ^b^	2510	9.47
**Hospital Death**		
No	23,316	88.0
Yes	3192	12.0
**30-day Readmission**		
No	23,549	88.85
Yes	2956	11.15

*NUTS II* Nomenclatura de Unidades Territoriais para Fins Estatísticos, nível II (Nomenclature of territorial units for statistics, 2 level); *R.A.* Região Autónoma (Autonomous Region)

^a^ National Health Insurance

^b^ transfer for conducting exams or follow up or lack of resources or treatment of associated condition

**Fig. 2:**
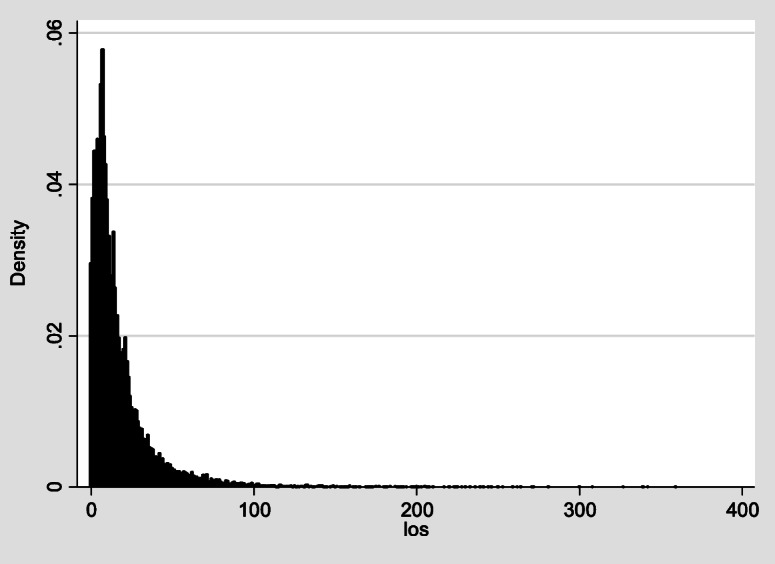
Length of stay distribution

The median LOS in our study was 11 days (IQR: 6–22). Figure 2 shows our dependent variable as zero-inflated, positively skewed, and over-dispersed with a mean of 17.9 days and a standard deviation of 22.4 days, indicating an obvious difference between the mean and median LOS. Moreover, to confirm overdispersion, results from the likelihood ratio test were significant, (LR χ2(1) = 0.79, *P* < 0.001), indicating overdispersed data. Therefore, the NBRM is more appropriate to analyze LOS when compared to PRM. The second stage in the second phase of our analysis, which was dedicated to evaluating the excessive zero counts, shows that 784 participants were with zero values representing around 3.0% of the total admissions. Results from the Vuong test confirm the significant favorability of using the NBRM over the PRM (z = 4.55, *p* < 0.001).

**Fig. 3:**
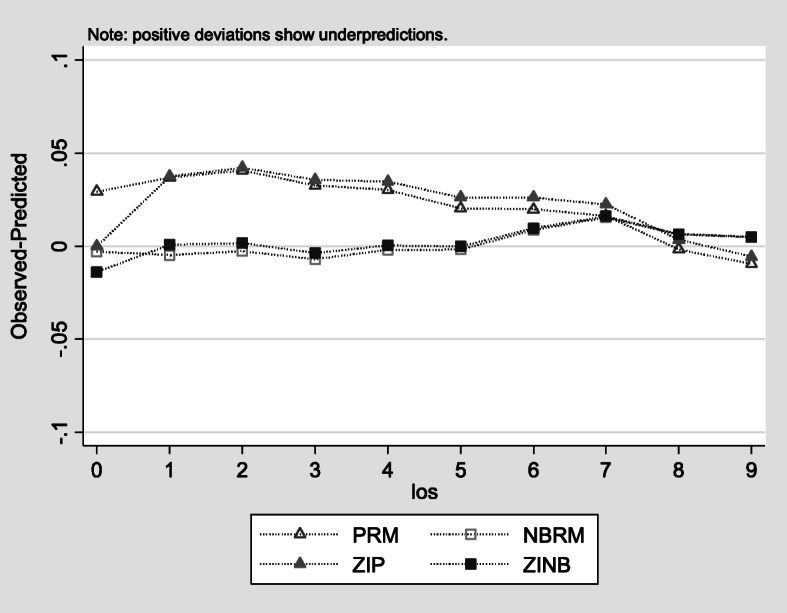
Comparisons among observed versus predicted probabilities among count models (*N* = 26,505). PRM – Poisson Regression Model; NBRM – Negative Binomial Regression Model, ZIP – Zero-inflated Poisson; ZINB – Zero-inflated Negative Binomial

Results from the “countfit” estimator in STATA show that NBRM and ZINB provide the best relative fit as they exhibited the least difference between the predicted and observed values and thus are preferred over the PRM and ZIP (Fig. 3). According to the figure, the best models are the models with estimates close to the zero line, the two negative binomial models. Being close to the zero line means that the predicted and the objected values are close to each other. Under the same countfit command, results from the AIC and the BIC tests (Table 2) illustrate a notable reduction in the AIC and BIC measures of both the NBRM (198,008.241 and 198,352.015, respectively) model and ZINB model (197,424.156 and 198,103.519, respectively) which means better quality of models compared to the PRM (445,042.083 and 445,377.671, respectively) and ZIP (432,277.401 and 432,948.578, respectively). Accordingly, the NBRM and ZINB were the models of choice since they demonstrate the lowest difference between the predicted and observed estimates and the lowest AIC and BIC test results.

**Table 2:** Tests and Fit Statistics (see the table in: Predicting length of stay from an electronic patient record system: a primary total knee replacement example)

PRM		BIC = 445,377.671	AIC = 445,042.083	Prefer	Over	Evidence
vs	NBRM	BIC = 198,352.015 AIC = 198,008.241 LR^a^	dif = 247,025.656 dif = 247,033.841 prob. = 0.000	NBRM NBRM NBRM	PRM PRM PRM	Very strong *p* = 0.000
vs	ZIP	BIC = 432,948.578 AIC = 432,277.401 Vuong^b^	dif = 12,429.093 dif = 12,764.682 prob. = 0.000	ZIP ZIP ZIP	PRM PRM PRM	Very strong *p* = 0.000
vs	ZINB	BIC = 198,103.519 AIC = 197,424.156	dif = 247,274.153 dif = 247,617.927	ZINB ZINB	PRM PRM	Very strong
NBRM		BIC = 198,352.015	AIC = 198,008.241	Prefer	Over	Evidence
vs	ZIP	BIC = 432,948.578 AIC = 432,277.401	dif = −2.346e+ 05 dif = − 2.343e+ 05	NBRM NBRM	ZIP ZIP	Very strong
vs	ZINB	BIC = 198,103.519 AIC = 197,424.156 Vuong^b^	dif = 248.497 dif = 584.085 prob. = 0.000	ZINB ZINB ZINB	NBRM NBRM NBRM	Very strong *p* = 0.000
ZIP		BIC = 432,948.578	AIC = 432,277.401	Prefer	Over	Evidence
vs	ZINB	BIC = 198,103.519 AIC = 197,424.156 LR^a^	dif = 234,845.060 dif = 234,853.245 prob. = 0.000	ZINB ZINB ZINB	ZIP ZIP ZIP	Very strong *p* = 0.000

*PRM* Poisson Regression Model, *NBRM* Negative Binomial Regression Model, *ZIP* Zero-inflated Poisson, *ZINB* Zero-inflated Negative Binomial

^a^ Verified with a likelihood ratio test

^b^ Verified with the Vuong test

Table 3 presents the predictors of LOS according to the ordinary least square model, the four selected count models, and the random-effects model. The OLS model results produced values that are overstating the actual coefficients compared to the count models. Regarding the negative binomial model with random-effects, admissions among males or admissions associated with TB infection, pneumocystis, cytomegalovirus, candidiasis, toxoplasmosis, or mycobacterium disease exhibit a highly significant increase in LOS. Moreover, perfect trends were observed in which a higher number of diagnoses or procedures lead to significantly higher LOS. On the other hand, admissions associated with Hepatitis C, HIV as the primary diagnosis, asymptomatic HIV, recurrent pneumonia, or weight loss show a statistically significant LOS decrease. Lower LOS was also observed among admissions registered in Norte, Centro, or Lisbon regions. Moreover, similar findings were observed among hospitalizations associated with exited against medical advice, a transfer to another hospital, inpatient mortality, or hospitalizations that occurred in the years that followed signing the financial bailout program (after 2011). As expected, the standard error in the ordinary count models was underestimated when compared to the random-effects count regression model.

**Table 3:** Regressions on Length of Stay (*N* = 26,505)

	Linear Regression (OLS)	Negative Binomial Regression	Poisson Regression	Zero-inflated Poisson Regression	Zero-inflated Negative Binomial	Random-effects Model
**Gender**						
Male	(Ref)	(Ref)	(Ref)	(Ref)	(Ref)	(Ref)
Female	−1.21*** (0.28)	−0.07*** (0.01)	− 0.07*** (0.00)	− 0.07*** (0.00)	− 0.08*** (0.01)	− 0.07*** (0.01)
**Age**						
18–29	(Ref)	(Ref)	(Ref)	(Ref)	(Ref)	(Ref)
30–39	0.06 (0.58)	0.00 (0.03)	− 0.00 (0.01)	0.00 (0.01)	0.01 (0.03)	−0.02 (0.03)
40–49	0.66 (0.57)	0.05* (0.02)	0.03*** (0.01)	0.03*** (0.01)	0.05** (0.02)	0.02 (0.03)
50–59	−0.01 (0.60)	0.01 (0.03)	−0.00 (0.01)	− 0.00 (0.01)	0.01 (0.03)	− 0.00 (0.03)
60–69	−1.07 (0.68)	− 0.03 (0.03)	− 0.07*** (0.01)	− 0.07*** (0.01)	− 0.03 (0.03)	−0.04 (0.04)
> =70	−1.64* (0.76)	− 0.06* (0.03)	− 0.09*** (0.01)	− 0.08*** (0.01)	−0.05* (0.03)	− 0.07* (0.04)
**Region of residence (NUTS II)**						
Non resident	(Ref)	(Ref)	(Ref)	(Ref)	(Ref)	(Ref)
Norte	−5.55*** (1.09)	−0.35*** (0.05)	−0.30*** (0.01)	− 0.30*** (0.01)	−0.35*** (0.05)	− 0.34*** (0.07)
Centro	−1.68 (1.12)	−0.13*** (0.05)	− 0.09*** (0.01)	−0.11*** (0.01)	− 0.14*** (0.05)	−0.28*** (0.07)
Lisbon	−2.50* (1.07)	−0.17*** (0.05)	−0.13*** (0.01)	− 0.13*** (0.01)	−0.18*** (0.05)	− 0.20*** (0.06)
Alentejo	−0.16 (1.53)	− 0.09 (0.07)	0.00 (0.02)	0.01 (0.02)	−0.10 (0.07)	− 0.15 (0.11)
Algarve	3.31** (1.19)	0.15*** (0.05)	0.20*** (0.01)	0.19*** (0.01)	0.14*** (0.05)	−0.06 (0.11)
**Years after economic crisis**						
No	(Ref)	(Ref)	(Ref)	(Ref)	(Ref)	(Ref)
Yes	−1.63*** (0.27)	−0.07*** (0.01)	−0.09*** (0.00)	− 0.09*** (0.00)	−0.07*** (0.01)	− 0.06*** (0.01)
**Having SNS**^a^						
Yes	(Ref)	(Ref)	(Ref)	(Ref)	(Ref)	(Ref)
No	−0.20 (0.74)	0.02 (0.03)	−0.00 (0.01)	− 0.01 (0.01)	0.01 (0.03)	0.03 (0.04)
**HIV as a primary diagnosis**						
No	(Ref)	(Ref)	(Ref)	(Ref)	(Ref)	(Ref)
Yes	−0.44 (0.30)	− 0.05*** (0.01)	− 0.04*** (0.00)	− 0.04*** (0.00)	− 0.05*** (0.01)	− 0.04** (0.02)
**Asymptomatic HIV**						
No	(Ref)	(Ref)	(Ref)	(Ref)	(Ref)	(Ref)
Yes	−3.88*** (0.54)	−0.28*** (0.02)	−0.28*** (0.01)	− 0.25*** (0.01)	−0.27*** (0.02)	− 0.27*** (0.03)
**Hepatitis B**						
No	(Ref)	(Ref)	(Ref)	(Ref)	(Ref)	(Ref)
Yes	0.19 (0.61)	0.01 (0.03)	−0.00 (0.01)	0.00 (0.01)	0.01 (0.03)	0.03 (0.03)
**Hepatitis C**						
No	(Ref)	(Ref)	(Ref)	(Ref)	(Ref)	(Ref)
Yes	−2.74*** (0.30)	−0.16*** (0.01)	−0.16*** (0.00)	− 0.15*** (0.00)	−0.16*** (0.01)	− 0.15*** (0.02)
**Associated TB infection**						
No	(Ref)	(Ref)	(Ref)	(Ref)	(Ref)	(Ref)
Yes	9.40*** (0.48)	0.50*** (0.02)	0.43*** (0.00)	0.43*** (0.00)	0.50*** (0.02)	0.52*** (0.02)
**Pneumocystis**						
No	(Ref)	(Ref)	(Ref)	(Ref)	(Ref)	(Ref)
Yes	1.28* (0.50)	0.11*** (0.02)	0.06*** (0.01)	0.06*** (0.01)	0.10*** (0.02)	0.08*** (0.03)
**Cytomegalovirus**						
No	(Ref)	(Ref)	(Ref)	(Ref)	(Ref)	(Ref)
Yes	10.96*** (0.80)	0.34*** (0.03)	0.32*** (0.01)	0.32*** (0.01)	0.34*** (0.03)	0.31*** (0.04)
**Mycobacterium disease**						
No	(Ref)	(Ref)	(Ref)	(Ref)	(Ref)	(Ref)
Yes	2.21*** (0.50)	0.09*** (0.02)	0.10*** (0.01)	0.10*** (0.01)	0.09*** (0.02)	0.07*** (0.03)
**Candidiasis**						
No	(Ref)	(Ref)	(Ref)	(Ref)	(Ref)	(Ref)
Yes	4.81*** (0.35)	0.22*** (0.01)	0.20*** (0.00)	0.20*** (0.00)	0.22*** (0.01)	0.19*** (0.02)
**Toxoplasmosis**						
No	(Ref)	(Ref)	(Ref)	(Ref)	(Ref)	(Ref)
Yes	10.31*** (0.58)	0.46*** (0.02)	0.40*** (0.01)	0.39*** (0.01)	0.46*** (0.02)	0.45*** (0.03)
**Kaposi Sarcoma**						
No	(Ref)	(Ref)	(Ref)	(Ref)	(Ref)	(Ref)
Yes	0.31 (0.66)	−0.01 (0.03)	0.00 (0.01)	0.01 (0.01)	−0.01 (0.03)	−0.02 (0.03)
**Recurrent Pneumonia**						
No	(Ref) -	(Ref)	(Ref)	(Ref)	(Ref)	(Ref)
Yes	7.90*** (1.49)	−0.44*** (0.07)	− 0.46*** (0.02)	− 0.47*** (0.02)	−0.44*** (0.07)	− 0.46*** (0.08)
**Weight loss**						
No	(Ref)	(Ref)	(Ref)	(Ref)	(Ref)	(Ref)
Yes	−3.21*** (0.70)	−0.15*** (0.03)	−0.15*** (0.01)	− 0.15*** (0.01)	−0.15*** (0.03)	− 0.12*** (0.04)
**Number of diagnosis**						
< =5	(Ref)	(Ref)	(Ref)	(Ref)	(Ref)	(Ref)
6–7	2.54*** (0.35)	0.19*** (0.02)	0.19*** (0.00)	0.17*** (0.00)	0.18*** (0.02)	0.19*** (0.02)
8–10	5.17 (0.36)	0.35*** (0.02)	0.34*** (0.00)	0.32*** (0.00)	0.34*** (0.02)	0.36*** (0.02)
> 10	10.47 (0.40)	0.55*** (0.02)	0.56*** (0.00)	0.54*** (0.00)	0.54*** (0.02)	0.55*** (0.02)
**Number of procedures**						
< =5	(Ref)	(Ref)	(Ref)	(Ref)	(Ref)	(Ref)
6–8	2.54*** (0.34	0.27*** (0.01)	0.25*** (0.00)	0.21*** (0.00)	0.25*** (0.01)	0.34*** (0.02)
9–11	5.36*** (0.37	0.45*** (0.02)	0.44*** (0.01)	0.39*** (0.01)	0.44*** (0.02)	0.54*** (0.02)
> 11	14.37*** (0.39	0.82*** (0.02)	0.79*** (0.00)	0.74*** (0.00)	0.80*** (0.02)	0.94*** (0.02)
**Emergency Admission**						
No	(Ref)	(Ref)	(Ref)	(Ref)	(Ref)	(Ref)
Yes	−0.76* (0.34)	−0.01 (0.01)	−0.05*** (0.00)	−0.06*** (0.00)	−0.02 (0.01)	0.02 (0.02)
**30-day Readmission**						
No	(Ref)	(Ref)	(Ref)	(Ref)	(Ref)	(Ref)
Yes	−1.41*** (0.39)	−0.03** (0.02)	−0.07*** (0.00)	− 0.06*** (0.00)	−0.03* (0.02)	− 0.01 (0.02)
**Type of intervention**						
Medical	(Ref)	(Ref)	(Ref)	(Ref)	(Ref)	(Ref)
Surgical	9.67*** (0.54)	0.34*** (0.02)	0.41*** (0.01)	0.41*** (0.01)	0.36*** (0.02)	0.32*** (0.03)
**Mode of transfer**						
No transfer	(Ref)	(Ref)	(Ref)	(Ref)	(Ref)	(Ref)
Transferred ^b^	−6.63*** (0.57)	−0.32*** (0.02)	−0.35*** (0.01)	−0.33*** (0.01)	− 0.32*** (0.02)	−0.35*** (0.04)
**Destination after discharge**						
Home	(Ref)	(Ref)	(Ref)	(Ref)	(Ref)	(Ref)
Another health service ^c^	6.31*** (0.68)	0.20*** (0.03)	0.30*** (0.01)	0.34*** (0.01)	0.21*** (0.03)	0.22*** (0.04)
Exit against medical advice	−4.73*** (0.63)	−0.44*** (0.03)	−0.42*** (0.01)	−0.35*** (0.01)	− 0.43*** (0.03)	−0.43*** (0.03)
In hospital death	−0.70 (0.39)	−0.07*** (0.02)	− 0.05*** (0.00)	0.01** (0.00)	− 0.06*** (0.02)	−0.11*** (0.02)
**Merged hospitals**						
No	(Ref)	(Ref)	(Ref)	(Ref)	(Ref)	(Ref)
Yes	−3.29*** (0.31)	−0.19*** (0.01)	−0.17*** (0.00)	− 0.17*** (0.00)	−0.19*** (0.01)	− 0.09 (0.09)
Constant	13.33 (1.37)	2.43*** (0.06)	2.47*** (0.02)	2.54*** (0.02)	2.46*** (0.06)	2.38*** (0.09)
Observations	26,505	26,505	26,505	26,505	26,505	26,505
Number of groups						54

Standard errors in parentheses

*NUTS II* Nomenclatura de Unidades Territoriais para Fins Estatísticos, nível II (Nomenclature of territorial units for statistics, 2 level); *R.A.* Região Autónoma (Autonomous Region)

*** *p* < 0.01, ** *p* < 0.05, * *p* < 0.1

^a^ National Health Insurance

^b^ transfer for conducting exams or follow up or lack of resources or treatment of associated condition

^c^ special service includes Home service, Specialized aftercare, Palliative care, long-term hospital care

**Fig. 4:**
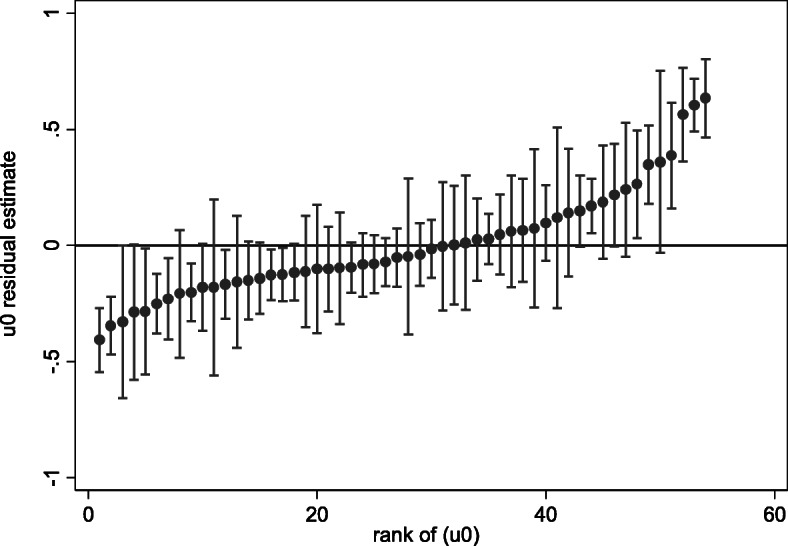
Caterpillar plot of the hospital effects

Fig. 4 represents the caterpillar plot of the hospital effects. The plot illustrates the random-effects model for 54 hospitals included in our study that was used to determine unmeasured and unobserved factors specific to each hospital with their respective 95% CI. The first nine hospitals have random-effects and respective 95% CI below zero, being considered with more quality when compared to the mean, whereas the last seven hospitals’ random-effects exhibit higher LOS (random-effects and corresponding 95%CI above zero). The plot shows that the remaining hospitals, almost 70% of the hospitals included, cannot be distinguished from the overall average.

## Discussion

This paper provides an illustration of statistical techniques that are appropriate to overcome obstacles linked to the prediction of count data with overdispersion using longitudinal predictors of LOS among HIV hospitalizations in Portugal. The advantages of the selected count models were presented and explained. To the best of our knowledge, this is the first study to consider analyzing LOS among HIV patients in Portugal by using the best count fit model after comparing the four aforementioned models.

The fact that applying different statistical techniques results in different results demonstrates the importance of precisely selecting a model that accurately represents the observed count data. In other words, we can say that this paper illustrates the consequences of using methods that do not precisely consider the nature of the data distribution. Analyzing LOS among HIV patients as a count variable with overdispersion will yield more precise outcomes if the assumptions behind the selected model are not violated.

Previous studies in Portugal that analyzed LOS among HIV patients either used logistic regression [28] or Poisson regression [29] without explaining the theory and assumptions behind selecting the Poisson regression over the other count models. LOS as an overdispersed count variable is violating the assumption of using the Poisson model in which the variance of LOS should be equal to the mean. As shown in our results, linear regression through the OLS technique, whose assumptions were violated by the count distribution of LOS data, produced values that are overstating the actual coefficients compared to the count models. Moreover, the same linear model identified some factors (emergency admissions, merged hospitals, and 30-day readmission) as significantly decreasing LOS although they are not when compared to the most appropriate models, namely ZINB and the random-effects model. On the contrary, the linear model showed a non-significant decrease in LOS among admissions with HIV as a primary diagnosis, while the ZINB and random-effects model showed a significant decrease. In addition, and when compared to the zero-inflated negative binomial model or the random-effects model, the other count models underestimated the standard errors of regression coefficients. However, this result was expected, especially for the random-effects model, since accurate standard errors will be generated if variation at multilevel, patient, and hospital levels is allowed in the analysis [20, 25]. Ignoring the hierarchy in multilevel data will result in data that underestimate the magnitude of hospital level’s standard errors of regression coefficients [20, 25]. The other technical problem is inflation in the number of independent observations at the hospital level of the hierarchy. For example, Poisson and ZIP identified some factors as significantly decreasing LOS, although they are not when compared to the random-effects model, namely 30-day readmission, merged hospitals, and emergency admissions. Moreover, all the included models showed a significant increase in LOS for admissions in Algarve, while the random-effects model, the most appropriate model, showed a non-significant decrease in LOS in the Algarve region. These findings can explain how using an inappropriate model may mislead policy making decisions.

The median LOS of 11 days in our study is equal to the median reported by a similar study in Brazil [39]. Shorter LOS was reported by similar studies in Spain (8 days) [40], United Kingdom (7.5 days) [41], and the United States (6 days) [42]. Moreover, the median of 11 days in Portugal is higher than the national Portuguese LOS average of 9 days [43]. On the contrary, higher LOS was reported in Colombia (14 days) [44], and Italy (16 days) [41]. Differences in LOS duration between countries can be explained by different applied policies on HIV infection control or different population samples among countries. This study pointed out several factors that tend to push LOS further after using the random-effects count model. First, socio-demographic factors indicate that admissions among females are frequently less prolonged than in men. This result is in accordance with a previous study in Ontario, Canada in which female sex was predictive of shorter hospital stays among HIV hospitalizations when compared to men [45]. This finding can be explained by the fact that male patients who are living with HIV carry a substantial burden of the disease with respect for severity [46], and morbidity [47], which can be reflected on LOS. Also, individuals who are registered as non-residents or individuals who are not registered in the National Portuguese Health System tend to have a significantly higher LOS. Since all Portuguese citizens and documented migrants are entitled to the NHS [48], this finding sheds light on how the migration status can affect health outcomes, especially among undocumented migrants in Portugal. Exit against medical advice was significantly associated with shorter hospital stays in our study. In general, exit against medical advice is associated with shorter stays and lower hospital charges at the beginning, as reported in previous studies [49, 50]. However, while this result is logical and expected, patients who exit against medical advice encounter a later higher risk of readmissions as shown in our previous study among HIV hospitalizations [26] or studies from other countries but among similar hospitalizations [51]. Accordingly, history of exit against medical advice can increase future utilization of healthcare resources [52]. Given this implication, further analysis of populations at risk and factors that can push exit against medical advice is mandatory to consider proper interventions.

Comorbidities were among the most important factors that tend to push LOS further. Also, comorbidities showed a perfect trend in which the higher number of comorbidities, diagnosis, or procedures were associated with higher LOS in days. Similar findings have been reported by a similar study in the United States in which the presence of comorbid conditions was a strong predictor of LOS [53]. The same study also defined the number of diagnoses and procedures as independent predictors of LOS [53]. Another study in Italy came to the conclusion that chronic diseases and comorbidities increase the costs of hospitalizations among HIV admissions [54]. Moreover, the presence of co-infections or AIDS-defining illnesses that are commonly associated with HIV patients led to a significant increase in LOS in our study. In general, these co-infections or AIDS-defining illnesses represent a major public health concern as they lead to patients’ disabilities and adverse hospital outcomes [44, 55–60]. These results from our study generally agree with those obtained in previous studies in other countries. For example, LOS was found to be significantly higher among individuals with HIV-TB coinfection in a study conducted in the United States [61]. Another study found that LOS and hospitalization costs were higher in the Hepatitis B-HIV co-infected patients compared to the Hepatitis B mono-infected patients or HIV mono-infected patients with statistically significant results [62]. Moreover, previous studies conducted in different countries defined Hepatitis C-HIV- co-infection as a significant predictor of LOS and early readmissions among HIV admissions [40, 41, 63]. In addition, another study defined the presence of an AIDS-defining illness as independent predictors of LOS [53]. In our earlier work [26], comorbidities and co-infections were among the main factors to increase the probability of 30-day readmission among HIV patients in Portugal. Since comorbidities and coinfections tend to increase both inpatient LOS and 30-day readmission, further exploration of any flaws in outpatient care and continuity of care following discharge should be addressed. In other words, to ensure more efficient care, healthcare providers should guarantee the entire care continuum for improvements. By encouraging an overall patient care plan, providers can potentially improve health outcomes while at the same time reducing costs. Accordingly, it is in the hospitals’ best interest to encourage communication, coordination, and follow-up with the primary care, rehabilitation centers, outpatient care, specialists, and general practitioners who are following their patients, even after they are discharged. In accordance with previous studies, HIV admissions associated with surgical interventions in our study were predictive of longer inpatient stays. These studies demonstrated that HIV/AIDS patients are more likely to develop surgical site infections and complications compared to the general population, hence, have longer LOS [64–66].

Years that followed the economic crisis in Portugal and culminated in signing the Economic Adjustment Program for Portugal, also known as the Bailout program, were associated with a significant decrease in LOS. Two facts can explain this phenomenon. First, by signing this Bailout program, Portugal was obligated to implement strict austerity measures that had led to a reduction of spending on sensitive health sectors, payments to NHS hospitals, day cases, and in-patients’ admissions [3, 26, 67]. These measures have reduced hospital quality by reducing LOS and number of admissions or by substituting in-patient hospitalizations by day cases [3, 26, 67]. The second fact that can explain this phenomenon is the continuous reforms of the hospitals’ sector in Portugal that translated into a 3 years plan for hospitals reforming that have been monitored by the Regional Health Authority [26, 68]. These reforms that started in 2011 have positively affected quality levels, with a significant reduction in LOS [26, 68].

The random-effects term included in our model and refers to unexplained factors specific to each hospital revealed obvious differences in quality among the hospitals included in our study. In other words, LOS is still significantly higher in some hospitals than others in Portugal. The seven hospitals that showed significantly higher LOS requires further investigation. These hospitals’ unexplained factors can range from discrepancies in medical expertise, health care, and human medical resources. However, this finding can be explained by how the health care system and the provided services can be affected by the socio-demographic inequalities in Portugal. These inequalities translate into an unfair distribution of medical resources, equipment, doctors, and nurses [69]. Moreover, some geographical areas in Portugal, particularly the coastal regions, exhibit higher concentrations of young populations and better economic growth indicators, leading to better health outcomes [69]. These findings necessitate a further examination of any potential discrepancies in the hospital’s performance by region that can be associated with higher LOS.

This study used the entire Portuguese national data for public hospital admissions, from which HIV patients were analyzed. The main strengths of our study can be concluded in the following points. First, we used a large dataset representing the entire national Portuguese admissions in public hospitals. Second, our study provided a comprehensive review and comparison between statistical procedures for analyzing count data to select the most optimal model. Third, we used multilevel random effect predictions that can help in the identification of outstanding hospitals and may serve as an indicator to assess hospital quality/performance. Finally, the study provides information on indicators that can push LOS further, which can enlighten health policymakers to control unnecessary hospitalizations. The main limitation of our study is the absence of additional socio-economic factors, access to primary care indicators, and clinical factors such as immune status, CD4 cell count, and ART at discharge that may contribute to a better understanding of factors that can increase LOS. Moreover, LOS could be underestimated due to death occurring during admission and/or in-hospital stay. Findings from this study support the conclusion of the Portuguese Court of Auditors, stating that the techniques used to analyze LOS within the DRG system is insufficient and is not the most appropriate way [70]. Accordingly, using fairly recent statistical techniques presented in our study to address complexly distributed data, unique problems associated with the prediction of LOS can be solved. Moreover, our study supports the Portuguese policy of cost reduction by preventing unnecessary spending [26]. Accordingly, LOS, as a quality/performance indicator, should be handled as a national priority, and health policies should be directed to consider addressing the determinants that can push it further.

## Conclusion

Analyzing LOS among HIV patients will yield more precise results if the nature of LOS as a count outcome met the assumptions behind the used statistical method. Using suboptimal techniques can mislead health professionals and policymakers. Results from our study can help to target the factors that tend to increase length of stay among HIV patients in Portugal and hence reduce unnecessary spending, given the weight and the economic burden of HIV/AIDS hospitalizations.

## Data Availability

Not applicable.

## Abbreviations

ACSS: Administração Central do Sistema de Saúde [Central Administration of the Health System]
AIC: Akaike information criterion
AIDS: Acquired Immunodeficiency syndrome
AP–DRGs version 21: All Patients Diagnosis Related Groups version 21
ART: Antiretroviral therapy
BIC: Bayesian information criterion
CI: Confidence intervals
DRG: Diagnosis Related Groups
HIV: Human immunodeficiency virus
ICD: International Classification of Diseases
IQR: Interquartile range
LOS: Length of stay
LR: likelihood-ratio
MDC: Major Diagnostic Category
NBRM: Negative binomial regression model
NHS: National Health Service (Portugal)
OLS: Ordinary Least Squares
PLWHA: People living with HIV/AIDS
PRM: Poisson regression model
SNS: Serviço Nacional de Saúde [National Health Services Coverage]
TB: Tuberculosis
ZINB: Zero-inflated negative binomial
ZIP: Zero-inflated Poisson
